# Local Droplet Etching‐Assisted Quantum Dot Epitaxy for Telecom C‐Band Quantum Light Emitters

**DOI:** 10.1002/nap2.70310

**Published:** 2026-10-16

**Authors:** Karolina E. Połczyńska, Paweł Wyborski, Michał Gawełczyk, Shima Kadkhodazadeh, Battulga Munkhbat, Stefano Sanguinetti, Elizaveta Semenova

**Affiliations:** ^1^ DTU Electro Technical University of Denmark Kongens Lyngby Denmark; ^2^ NanoPhoton‐Center for Nanophotonics Technical University of Denmark Kongens Lyngby Denmark; ^3^ Institute of Theoretical Physics Wrocław University of Science and Technology Wrocław Poland; ^4^ DTU Nanolab Technical University of Denmark Kongens Lyngby Denmark; ^5^ Department of Materials Science University of Milano‐Bicocca Milano Italy

**Keywords:** local droplet etching epitaxy, quantum dots, telecom C‐band

## Abstract

Significant progress in quantum light sources for quantum communication applications requires reproducible and symmetric quantum emitters acting as single‐photon sources capable of generating entangled photons on demand at specific telecom wavelengths. Here, we propose telecom‐emitting epitaxial quantum dots (QDs) fabricated by metal‐organic vapor phase epitaxy (MOVPE) using the local droplet etching (LDE) approach. The resulting well‐defined, low‐density ($10^{9}$/${cm}^{2}$) QDs based on ${\text{In}}_{x}{\text{Ga}}_{1-x}\text{As}$ are formed in symmetric LDE nanoholes in ${\text{In}}_{0.52}{\text{Al}}_{0.48}\text{As}$. Detailed transmission electron microscopy provides comprehensive insight into the structural properties of the QDs. Photoluminescence spectroscopy reveals single QD emission lines (linewidth ∼0.2 meV, fine structure splitting ∼60 μeV) with negligible impact of nonradiative processes, with decay ∼1−2 ns comparable to theoretical simulations, whereas second‐order autocorrelation measurements confirm clear single‐photon emission with $g^{\left(2\right)}\left(0\right)=0.07\pm 0.02$ under above‐band continuous‐wave excitation and $g^{\left(2\right)}\left(0\right)=0.16\pm 0.18$ under pulsed excitation. Precise numerical modeling, combining multiband k⋅p and configuration interaction methods, identifies thermal excitation pathways that explain the persistence of emission up to liquid‐nitrogen temperatures. These results highlight the versatility of the LDE approach for integrating new material systems and provide a promising foundation for future studies aimed at scalable fabrication of quantum light sources with tailored emission properties.

## Graphical abstract

InGaAs quantum dots emitting in telecom wavelengths are fabricated using local droplet etching in a MOVPE growth process. The resulting low‐density emitters exhibit well‐defined optical properties, demonstrating the potential of this approach for developing quantum‐dot sources across the telecom wavelength range.

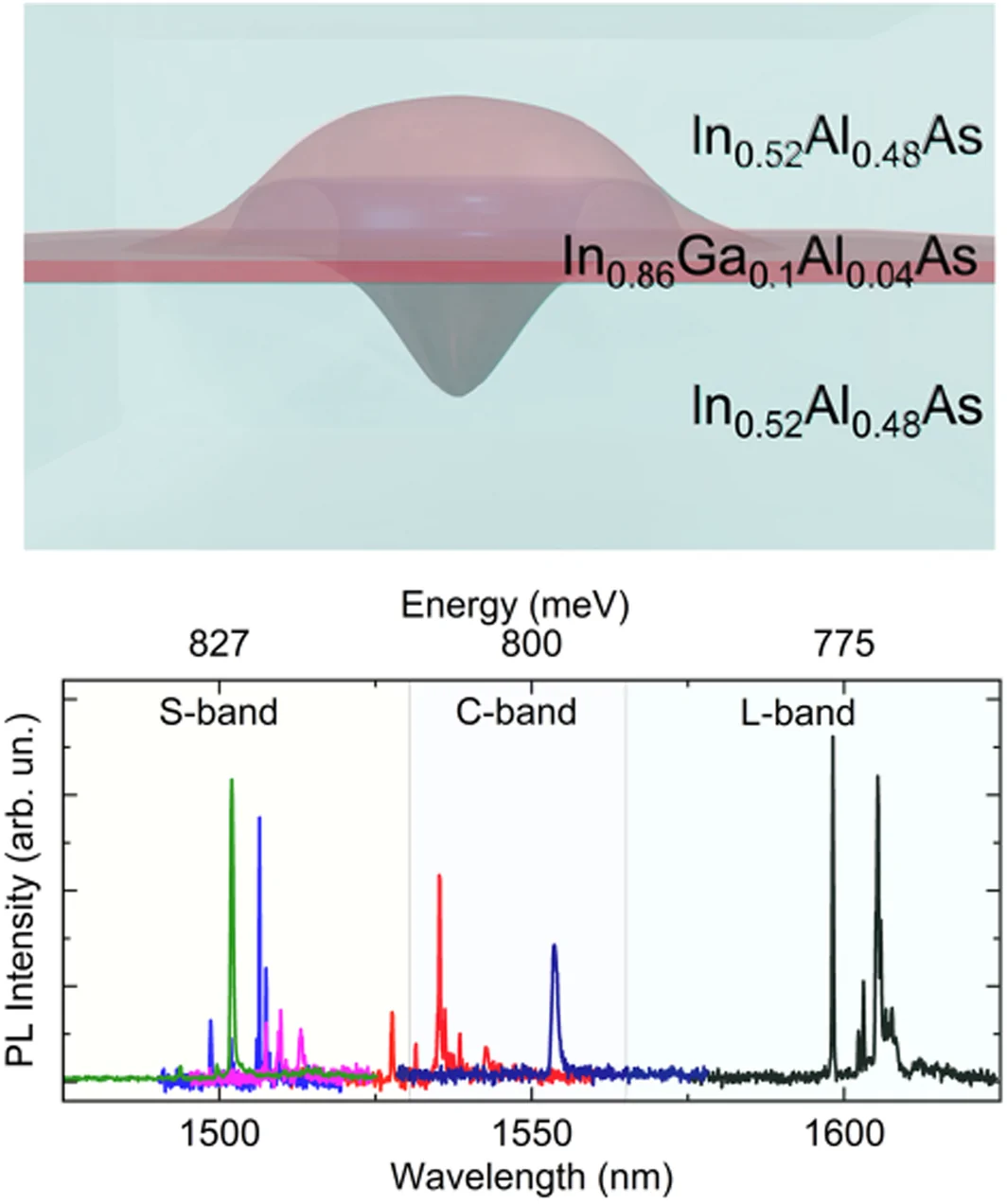

## 1 Introduction

Among solid‐state quantum light sources operating in the telecom wavelength range, epitaxial quantum dots (QDs) are considered one of the leading candidates for enabling large‐scale quantum communication and computing architectures [1]. In particular, applications such as quantum teleportation, quantum repeaters, and distributed quantum computing rely on sources of entangled photon pairs [2, 3, 4]. In semiconductor QDs, polarization‐entangled photon pairs can be generated via the biexciton–exciton radiative cascade [5, 6, 7]. A prerequisite for exploiting the biexciton–exciton cascade as a robust source of entanglement is suppressing the excitonic fine‐structure splitting (FSS) below the Fourier‐limited emission linewidth, enabling truly indistinguishable decay channels. Recent progress in epitaxial QDs emitting in C‐band has brought the FSS to values such as 61±31 μeV [8], 20–40 μeV [9], or 4.8±0.4 μeV [10] (values registered for InAs QDs). Generating polarization‐entangled photon pairs does not require strictly zero FSS, as values of a few microelectronvolts are generally sufficient to preserve a high degree of entanglement [11]. Without additional tuning, this can only be attained in QDs with a highly symmetric in‐plane potential, underscoring the importance of synthesizing emitters with near‐ideal lateral symmetry, combined with post‐growth methods for tuning FSS.

Several post‐growth approaches have been developed to tune the FSS in semiconductor quantum dots [12]. Thermal annealing can reduce the FSS from nearly 100 μeV to below 10 μeV by modifying the confinement potential, although at the expense of significantly weaker carrier confinement [13]. Electric fields enable in situ tuning over ranges approaching 90 μeV with vertical devices offering the advantage of maintaining high emission intensity [9, 14, 15]. Anisotropic strain provides continuous tuning by several tens of μeV and can be combined with electric or magnetic fields, whereas magnetic fields of several tesla can also reduce the FSS to values suitable for entangled‐photon generation [2, 10, 16, 17]. Despite their effectiveness, these approaches require additional external control, making the direct growth of quantum dots with intrinsically low FSS a key issue.

The most common are QDs grown via the Stranski–Krastanov strain‐driven mode [18, 19, 20, 21, 22] where a lattice mismatch defines the QD formation. Although this approach yields QDs with excellent optical performance, it has an inherent reduced in‐plane symmetry resulting from anisotropic indium surface diffusion during QD formation. This asymmetry leads to significant FSS. One of the promising approaches toward reducing FSS is the growth of Stranski–Krastanov QDs on GaAs(111)‐oriented substrates [23, 24, 25]. The inherent threefold rotational symmetry of the (111) surface reduces in‐plane anisotropy and suppresses exciton FSS, enabling the realization of quantum dots with improved photon indistinguishability and high entanglement fidelity [26]. In addition, the (111) orientation facilitates strain relaxation in metamorphic buffer layers [27], which is advantageous for extending the emission wavelength of In(Ga)As quantum dots toward telecommunication bands. However, this approach requires careful strain engineering and remains challenging due to the formation of strain‐induced defects and limited flexibility in achieving high‐quality quantum dots at longer wavelengths. To overcome those limitations, alternative growth mechanisms have been explored, such as droplet epitaxy (DE) [28, 29, 30].

DE is a highly versatile technique that enables the precise tailoring of nanostructures, allowing the fabrication of a wide variety of morphologies in situ during a single epitaxial growth process [28]. By carefully controlling parameters such as substrate temperature, annealing duration, ambient in the growth chamber, and the quantity of deposited material, it is possible to engineer nanostructures with a wide variety of shapes and properties [31, 32], such as single rings [33], concentric double rings [34], nanomounds [35], ring disc [36], and coupled dot ring structures [37]. In addition, DE provides high control over the areal density of structures with record‐low densities achieved [38].

One branch of DE is the so‐called local droplet etching (LDE) [39, 40, 41, 42, 43, 44]. LDE occurs during annealing of metal droplets at a specific substrate temperature and atom group V ambient overpressure inside the growth chamber, such that the liquid metal droplet locally dissolves the underlying compound semiconductor layer. The dissolved material migrates through the droplet and reprecipitates at the triple phase boundary where the liquid droplet, the solid epitaxial layer, and the gaseous ambient coexist. Simultaneously, a portion of the metal from the droplet migrates across the surface, combines with the group V atoms supplied in the chamber atmosphere, and crystallizes on the top of the surface [40]. This process results in the formation of an etched nanohole surrounded by a nanoring, which typically exhibits an elevated concentration of the droplet metal relative to the original etched epitaxial layer.

The LDE‐generated nanoholes are immediately suitable for subsequent infilling with a material of choice, facilitating the formation of strain‐independent QDs [45, 46, 47, 48]. By varying the amount and composition of the infilling material, the QD optical emission energy can be tuned [49], and complex heterostructures such as coupled nanostructures can be realized [37]. Typically, nanoholes formed by LDE, and also resulting QDs, exhibit a high degree of in‐plane symmetry compared to Stranski–Krastanov QDs, as the droplet‐induced etching process is governed predominantly by the nearly isotropic dissolution of the semiconductor material into the liquid metal droplet [50, 51, 52]. In contrast, the pronounced elongation observed in Stranski–Krastanov QDs along specific crystallographic directions (e.g., the [1, 2, 3, 4, 5, 6, 7, 8, 9, 10]) originates from anisotropic surface diffusion and facet‐dependent adatom incorporation during strain‐driven epitaxial growth [53]. These same anisotropies also influence the subsequent material redistribution around LDE‐etched pits, giving rise to nanorings with nonideal symmetry and distinct facets [44]. However, careful optimization of growth parameters, such as temperature and amount of applied metal [50, 54], can further improve the in‐plane symmetry of both the nanoholes and the surrounding nanorings. Fabricating highly symmetric nanoholes and subsequently filling them with QD material is a promising strategy for suppressing FSS of the excitonic states, facilitating the realization of polarization‐entangled photon emitters.

Here, we report the development of an epitaxial growth protocol and detailed characterization of the morphology and optical properties of low‐density ${\text{In}}_{y}{\text{Ga}}_{1-y}\text{As}$ QDs obtained by filling nanoholes in ${\text{In}}_{0.52}{\text{Al}}_{0.48}\text{As}$ formed via LDE. In contrast to the recently reported LDE quantum dots grown by MBE on GaAs (111)A substrate with a metamorphic buffer layer [55], our approach employs MOVPE and a conventional ${\text{In}}_{0.52}{\text{Al}}_{0.48}\text{As}$ barrier lattice matched to an InP (001) substrate. Although achieving telecom C‐band emission in this material platform requires increasing the indium content of the QDs, thereby introducing lattice mismatch with the barrier, we demonstrate that this challenge can be successfully overcome, resulting in high‐quality single‐photon emission in the telecom C‐band. The resulting QDs exhibit a characteristic two‐part morphology [56] consisting of a highly symmetric region defined by the nanohole geometry and a slightly asymmetric dome‐shaped top that arises from material accumulation and anisotropic adatom diffusion during the infilling and overgrowth steps. We perform numerical modeling that combines multiband k⋅p and configuration interaction methods based on transmission electron microscope (TEM) data to support the optical characterization. Experimentally confirmed FSS values are in the range of 40−70 μeV comparable to the spectral resolution of the setup. Our QDs exhibit average decay times of 1.345 ns and a degree of linear polarization (DOLP) of 9%, an activation energy of 4.6 meV, and $g^{\left(2\right)}\left(0\right)=0.07\pm 0.02$, proving single‐photon emission.

## 2 Morphology

The first step in LDE‐based QD synthesis is the preparation of nanoholes with a controlled surface density. The LDE mechanism occurs during annealing in the ${\text{AsH}}_{3}$ atmosphere after the deposition of metallic droplets on top of the semiconductor surface. During the development of the growth protocol, two types of droplets were tested: aluminum and indium. Despite many trials with different amounts of deposited material and annealing parameters, we have never reached the LDE regime with Al droplets. Low Al mobility indicates that high temperatures of annealing are required to observe LDE, and even after annealing at 750°C, we observed only droplet ripening. Higher temperatures would damage our sample, so we decided to proceed with another droplet material. 6.735 Å layer of In was deposited, forming metallic droplets on top of the ${\text{In}}_{0.52}{\text{Al}}_{0.48}\text{As}$ layer lattice‐matched to InP, followed by annealing in the ${\text{AsH}}_{3}$ ambient. During this annealing step, the underlying semiconductor layer is anisotropically etched. The atoms from the melted layer dissolve in the In droplet and diffuse to the surface where they crystallize into nanorings along the droplet circumference. The whole process is illustrated in Figure 1a–c.

**FIGURE 1:**
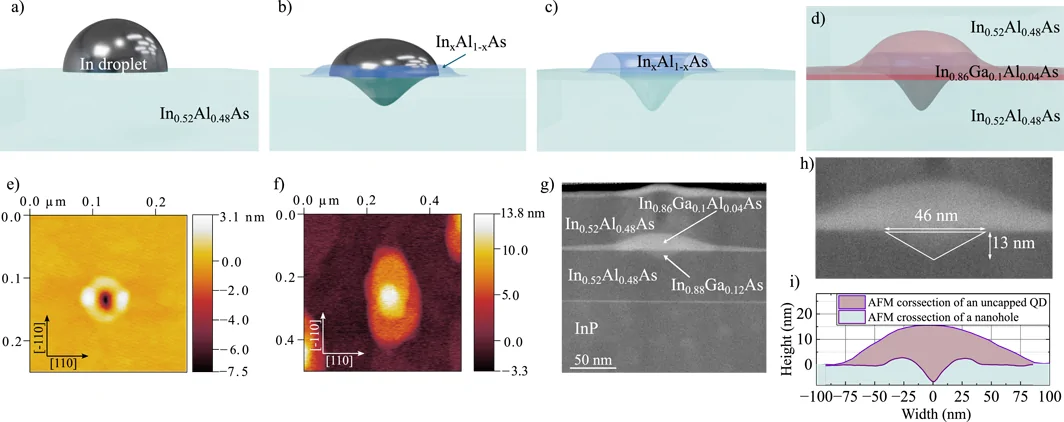
Schematic illustration of the QD growth based on the local droplet etching mechanism, demonstrated for our sample: (a) An indium droplet is deposited on the surface of a 50 nm layer of ${\text{In}}_{0.52}{\text{Al}}_{0.48}\text{As}$. (b) During annealing under an arsenic flux, the droplet locally melts the underlying layer, and the excess material crystallizes around the droplet, forming a nanoring. (c) Once the droplet is completely consumed, the nanohole and nanoring structures are fully developed. (d) The nanohole is then filled with an equivalent of 2 nm of ${\text{In}}_{y}{\text{Ga}}_{1-y}\text{As}$ to form a QD. Finally, a 50 nm ${\text{In}}_{0.52}{\text{Al}}_{0.48}\text{As}$ capping layer is grown. (e) Nanohole etched in the ${\text{In}}_{0.52}{\text{Al}}_{0.48}\text{As}$ layer by In droplets imaged by AFM. (f) Uncapped QDs: LDE nanohole filled with 2 nm of ${\text{In}}_{0.53}{\text{Ga}}_{0.47}\text{As}$ imaged by AFM. (g) HAADF STEM image of the cross section of a QD in the sample recorded along a <110> zone‐axis. The composition of each region was determined using EDS measurements (see: Supporting Information S1: Figure S2). (h) Close‐up of the QD with the marked filled nanohole area measuring 46 nm in diameter and 13 nm in depth, compared with (i) cross‐section of an empty nanohole and an uncapped QD, measured in the [110] direction with AFM, determined sizes of the nanohole: 27 nm diameter, 6.6 nm depth.

The AFM characterization of resulting LDE nanoholes etched in the ${\text{In}}_{0.52}{\text{Al}}_{0.48}\text{As}$ layer with a surface density of $∼10^{9}\;{\text{cm}}^{-2}$ is presented in Supporting Information S1: Figure S1a–c, and one exemplary case is depicted in Figure 1e. Statistical data for diameters based on 2×2 μm AFM images show that the mean nanohole diameter measured at the level of the plane surface is 30.5 nm in the X=[110] direction and 34.8 nm in the Y=[−110] direction, indicating their close‐to‐cylindrical symmetry with 1.14 aspect ratio. The high‐resolution measurement of an exemplary nanohole presented in Figure 1e reveals a depth of approx. 7.5 nm enough to allow QD height tunability via the amount of deposited QD material. The cross section (shown in Supporting Information S1: Figure S1c) shows slight elongation of the nanohole along [−110] and asymmetry in the nanoring height, which is 30% higher along [−110] than [110]. Different shapes of the nanohole and the nanoring associated with different crystallographic directions are standard for the LDE mechanism observed also for different materials [44, 52, 57].

On top of the ${\text{In}}_{0.52}{\text{Al}}_{0.48}\text{As}$ surface with etched nanoholes, we applied an equivalent of 2 nm (approx. 7 monolayers) of ${\text{In}}_{0.53}{\text{Ga}}_{0.47}\text{As}$. As shown in AFM studies of such a sample presented in Supporting Information S1: Figure S1d–f and Figure 1f, this amount of material overfilled the nanoholes. As a result, we observe growth not only inside the nanohole, but also above and around it with an average elongation of 31% in the [−110] direction. The height of uncapped QDs is 15 nm on average. The surface density of QDs is in agreement with the surface density of nanoholes ($∼10^{9}$ ${\text{cm}}^{-2}$).

As mentioned and presented in Figure 1d, the nanoholes were filled with nominally 2 nm of ${\text{In}}_{0.53}{\text{Ga}}_{0.47}\text{As}$. One could naively expect partial, flat‐terminated filling of the nanoholes with the material of designed composition. However, scanning transmission electron microscopy (STEM) characterization shown in Figure 1g,h, combined with energy‐dispersive X‐ray spectroscopy (EDS) analysis presented in Supporting Information S1: Figure S2, revealed a different shape and precise composition of the QDs. Nanoholes were completely filled and overgrown with ${\text{In}}_{0.88}{\text{Ga}}_{0.12}\text{As}$, indicating preferential In incorporation in the nanohole vicinity. The area surrounding the nanohole and corresponding to the nanoring consists of 4% Al (${\text{In}}_{0.86}{\text{Ga}}_{0.1}{\text{Al}}_{0.04}\text{As}$), indicating intermixing with the barrier and the QD material in that region. The 7‐monolayer layer is uniformly distributed, and EDS analysis indicates it is composed of ${\text{In}}_{0.55}{\text{Ga}}_{0.28}{\text{Al}}_{0.18}$As. In this case, the unexpected Al presence was probably registered as a result of the beam broadening in the EDS data.

Comparing STEM imaging (Figure 1h) of capped QDs with AFM of empty nanoholes and uncapped QDs (Figure 1i), we notice size differences. The size of the filled nanohole visible in the STEM image is almost twice that of the empty nanohole measured under AFM. Such a difference in those spatial parameters is mainly due to limitations in AFM measurements related to tip size, resulting in underestimated sizes of deep, narrow nanoholes. Another reason may be the intermixing between ${\text{In}}_{0.52}{\text{Al}}_{0.48}\text{As}$ layer and ${\text{In}}_{x}{\text{Ga}}_{1-x}\text{As}$ filling material that could effectively broaden the nanostructure.

Although the growth conditions employed in this study were optimized to support the formation of a uniform 2 nm layer of ${\text{In}}_{0.53}{\text{Ga}}_{0.47}\text{As}$ on an ideally flat surface, the prepatterned nature of our sample resulted in a significantly higher indium concentration within and above the filled nanoholes. This phenomenon can be attributed to the high mobility of indium atoms [58], which promotes the segregation of indium near the locally strained regions of the structure. Despite utilizing a nominally strain‐free material system, it was not possible to completely eliminate the strain in the resulting nanoring structures. By design, these structures inherently incorporate a higher indium content due to indium droplet consumption during the LDE process. Consequently, indium accumulation in these regions locally modifies the lattice constant, further enhancing the strain in these areas.

## 3 Theoretical Predictions

To guide the analysis of our experimental results and get more insight, we perform a detailed simulation of a QD. Uncapped QDs likely exhibit slight differences in morphology compared to capped QDs. Therefore, we use the morphology revealed in Figure 1g,h and Supporting Information S1: Figure S2. Our simplified model of a QD consists of an inverted cone (46 nm base diameter D, 13 nm height H) nominally filled with ${\text{In}}_{0.88}{\text{Ga}}_{0.12}\text{As}$, and a 2 nm‐thick layer followed by a dome‐shaped cap (80 nm base diameter $D_{d}$, 17 nm height $H_{d}$) of ${\text{In}}_{0.86}{\text{Ga}}_{0.1}{\text{Al}}_{0.04}\text{As}$, all embedded in the ${\text{In}}_{0.52}{\text{Al}}_{0.48}\text{As}$ barrier. To account for the normal diffusion of atoms at interfaces, this nominal material distribution is subject to Gaussian averaging with a spatial extent of 2 nm. In Figure 2a, we show the spatial distribution of the material expressed as the value of x in the (${\text{Al}}_{0.48}{\text{In}}_{0.52}\text{As}$)_1–*x*_ (${\text{Al}}_{0.06}{\text{Ga}}_{0.1}{\text{In}}_{0.84}\text{As}$)_*x*_ alloy.

**FIGURE 2:**
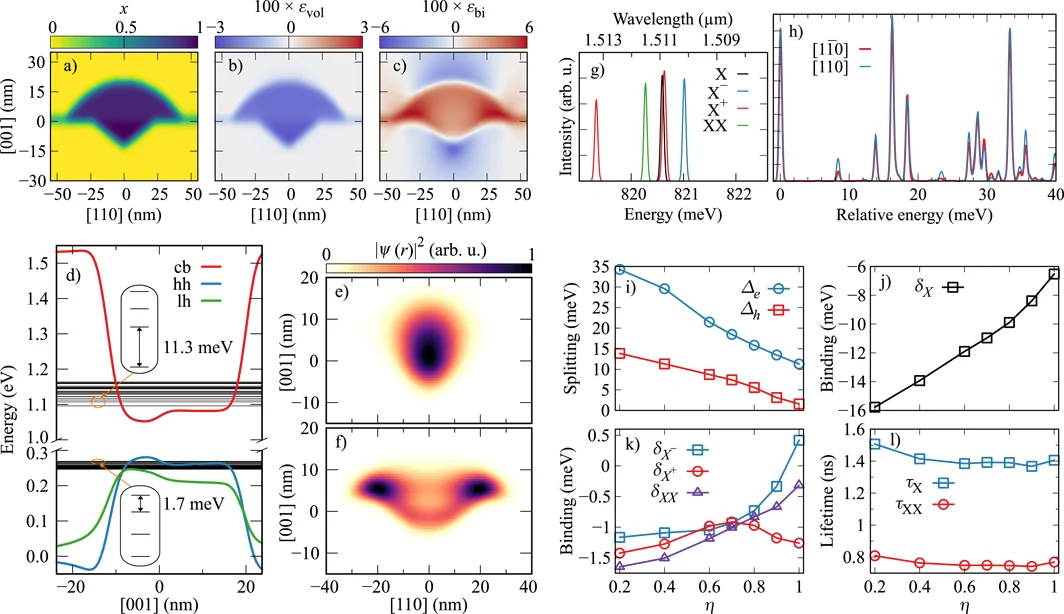
Theoretical predictions based on numerical calculations: Cross sections in the $\left(1\bar{1}0\right)$ plane of (a) material distribution, (b) volumetric strain $\varepsilon_{\text{vol}}=\sum_{i}\varepsilon_{ii}$, (c) biaxial strain $\varepsilon_{\text{bi}}=2\varepsilon_{zz}-\varepsilon_{xx}-\varepsilon_{yy}$ in the simulated QD. (d) Calculated single‐particle energy levels (horizontal lines) shown with the band edges of the conduction and valence bands along the [001] axis (through the middle of the QD). Insets show splittings of the lowest‐energy levels. (e) Probability density $|\Psi {|}^{2}$ for the electron ground state. (f) as in (e) but for the hole. (g) Calculated spectrum of the lowest‐energy optical transitions arising from electron‐hole recombination in excitons (black), positive (red) and negative trions (blue), and biexcitons (green). Note that two $X^{+}$ transitions fall within the range considered. (h) Simulated absorption‐like exciton spectrum for the two in‐plane linear polarizations; The energy is given relative to the ground‐state transition. (i)‐(l) Calculated dependence on the QD dome size of (i) electron (blue) and hole (red) lowest level splitting, (j) exciton binding energy, (k) trion and biexciton binding energies, and (l) exciton and biexciton ground state radiative lifetimes.

In Figure 2b,c, we show the distribution of the calculated volumetric and biaxial strains in the QD and its surroundings. The volumetric strain is low as expected for this nominally lattice‐matched material system. In turn, even low biaxial strain is important for hole confinement and heavy–light hole splitting in such large QDs. We notice its inhomogeneity within the QD with the highest values present in the external part of the dome.

The simulated energy levels for the modeled QD are shown in Figure 2d together with the conduction‐ (cb) and valence‐band (heavy‐hole hh, light‐hole lh) edges plotted along the growth axis through the middle of the QD. The single‐particle estimate for the optical transition energy is 826.7 meV (1.5 μm). Large QD volume results in relatively dense ladders of states with the lowest level splittings of $\Delta_{e}=11.3$ meV for electrons and $\Delta_{h}=1.7$ meV for holes. Figure 2e,f show the probability density for the electron and hole ground states. The electron is predominantly confined in the filled nanohole, but its wavefunction also extends vertically to the dome. On the other hand, the hole is mainly confined to the outer part of the dome with only partial overlap with the nanohole region. This specific hole confinement is due to the aforementioned biaxial strain distribution, which, via the deformation potential, creates an additional confining potential for heavy holes.

Calculation of few‐carrier states allows us to simulate the spectrum of ground‐state emission for the exciton, positive and negative trion, and biexciton shown in Figure 2g relative to the ground‐state exciton line at 820.6 meV (1.511 μm). We find that the formation of a negative trion is weakly unfavorable compared to an exciton, as its binding energy $\delta_{X^{-}}=E_{X^{-}}-\left(E_{X}+E_{e}\right)\;\approx \;0.4$ meV is positive where $E_{e}$ is the single‐particle electron ground‐state energy in the conduction band. A similar measure for the biexciton is low but negative, $\delta_{XX}=E_{XX}-2E_{X}\;\approx -0.3$ meV. We predict the positive trion to be most stable with $\delta_{X^{+}}=E_{X^{+}}-\left(E_{X}-E_{h}\right)\;\approx -1.25$ meV where $E_{h}$ is the single‐particle electron ground‐state energy in the valence band, as the large dome allows for avoidance of the two holes. Noticeably, a second peak related to the excited states of $X^{+}$ is predicted to lie very close to the exciton line.

Focusing on excitonic emission, we plot in Figure 2h a spectrum of optical transitions simulated by calculating the 2000 lowest‐energy exciton states with their lifetimes and polarizations, broadening each transition line by ℏ/τ (Fourier‐limited linewidth), and convoluting with a Gaussian with an arbitrary width of σ=0.2 meV to represent the total broadening due to laser linewidth, spectral diffusion, and phonon effects [59]. Bright lines are observed at approximately 16 meV, 18 meV, and 28–32 meV above the ground‐state transition. These transitions originate from exciton states dominated by specific electron‐hole configurations $e_{i}h_{j}$ where i and j denote single‐particle state numbers. The peak at 16 meV is due to a state dominated by the $e_{2}h_{3}$ configuration. The 18 meV peak involves two configurations, $e_{1}h_{2}$ and $e_{3}h_{2}$, whereas the peaks near 30 meV arise from highly mixed superpositions of various configurations that are difficult to characterize. Such a high level of configuration mixing is a fingerprint of a weaker exciton confinement regime where electron‐hole interactions significantly influence excitonic states.

Finally, we examine the dependence of the simulated QD properties on the dome size, the most variable morphological parameter. Such a study is important, as the single QDs probed with μPL are typically not the majority that yield the PL maximum but rather slight outliers. We vary the dome height and diameter uniformly as $\eta H_{d},\eta D_{d}$ (with η=1 reproducing the nominal modeled QD and η=0 corresponding to no dome). In Figure 2i, we show the dependence of single‐particle splittings on η. Although both carriers are affected, we observe a stronger dependence for holes with a ≈5.5‐fold change in the splitting already for a 1.7×change in the dome size (from η=1 to η=0.6). This is understandable in view of the hole confinement in the dome evidenced above. Tighter confinement increases the electron‐hole overlap and, as shown in Figure 2j, enhances the exciton binding energy $\delta_{X}=E_{X}-\left(E_{e}-E_{h}\right)$ where $E_{X}$ is the exciton ground‐state energy, and a negative value means a bound complex. Although the electron‐hole interaction becomes stronger, its relation to electron splitting does not change much, with $|\delta_{X}/\Delta_{e}|\;\approx \;0.45-0.62$. At the same time, a similar ratio for the hole decreases from $|\delta_{X}/\Delta_{h}|\;\approx \;4$ to ≈1 as η decreases, showing that with decreasing dome size, the confinement changes from intermediate toward a stronger confinement regime for the exciton. Reduced volume also enhances most of the binding energies of higher carrier complexes with their dependence on η shown in Figure 2k. Finally, we show in Figure 2l that, in contrast to other properties, the exciton and biexciton radiative lifetimes are very weakly changed as a function of η, which underlines the resilience of the basic optical QD properties to such morphology variation. This weak change in lifetimes can be understood as arising from an interplay between electron‐hole overlap, which is maximized for a small‐dome QD and the correlation‐induced enhancement of emission in a large‐dome QD [60]. It needs to be noted here that, as a result of finite single‐particle bases used in the many‐particle calculations, the binding energies can be expected to be underestimated and the lifetimes overestimated.

## 4 Optical Properties

To verify the optical properties of QDs and their potential for single‐photon emission, we performed optical measurements for the investigated sample at cryogenic temperatures using the μPL setup (see Methods). In addition to substrate and barrier‐layer emission, low‐spatial‐resolution measurements reveal separated single lines in the third telecommunication window (see Supporting Information S1: Figure S3), demonstrating the potential of this platform for realizing single‐QD emitters in the telecom range. The low number of QD lines supports a low surface density of QDs, equal to that of nanoholes $\approx \;10^{9}\;{\text{cm}}^{-2}$, providing good access to single‐QD investigations even in an unpatterned sample. More accurate high‐spatial‐resolution measurements reveal single‐QD emission in the range between 1480 and 1620 nm, as shown in Figure 3a. The analysis of their linewidth revealed an average broadening of about 200 μeV (from 134 to 489 μeV, as shown in Supporting Information S1: Figure S4) comparable to that of standard Stranski‐Krastanov InAs QDs emitting in the third telecom window [53, 61]. However, those values are still higher than state‐of‐the‐art values for third‐telecom‐range QDs below 50 μeV [62, 63], suggesting still a significant impact of charge noise from traps likely located at material interfaces and arising from residual‐strain and material‐redistribution‐driven defect formation.

**FIGURE 3:**
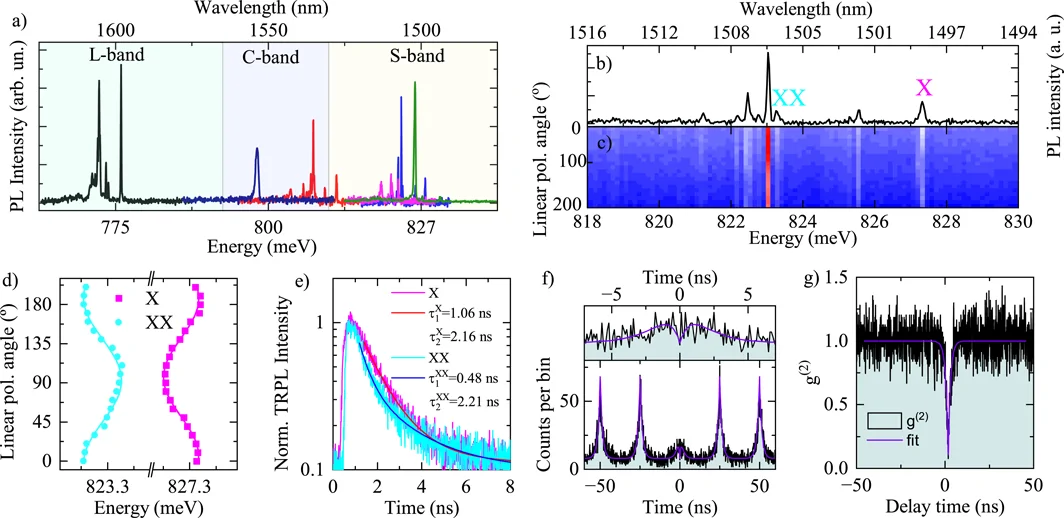
Optical properties of the LDE QDs. QD1: (a) Micro‐photoluminescence spectra of single QDs observed in different spots on the sample measured at 5 K under 650 nm continuous‐wave excitation. (b) μPL spectrum of an individual QD with identified excitonic complexes (exciton, X, and biexciton, XX). The biexciton binding energy is 4 meV. Measurement performed at 4 K under 650 nm excitation. (c) Polarization‐resolved μPL. (d) Sinusoidal fit to the polarization‐resolved data from panel b. FSS value obtained by the fitting: 69±9 μeV. (e) Time‐resolved photoluminescence for the X and XX lines with decay times $\tau_{1}^{X}=1.06\pm 0.26$ ns, $\tau_{2}^{X}=2.16\pm 3.35$ ns and $\tau_{1}^{XX}=0.48\pm 0.04$ ns, $\tau_{2}^{XX}=2.21\pm 0.18$ ns. QD2: (f) $g^{\left(2\right)}$ measured with pulsed excitation, (g) $g^{\left(2\right)}$ measured with CW excitation at 650 nm $g^{\left(2\right)}\left(0\right)=0.07\pm 0.02$.

The results demonstrate the realization of telecom C‐band quantum dots with confirmed single‐photon emission using the applied epitaxial QD growth approach. The more representative QD analyzed in detail in Supplementary Information (Supporting Information S1: Figure S5) shows good agreement with theoretical predictions based on the morphology revealed by STEM scans with QDs formed by a hole and a dome. However, the challenging emission‐line identification hinders verification of other QD properties in that case. Thus, we also focus on other QDs observed within the same structure, which are possibly less typical but show larger separations between excitonic lines.

Figure 3b presents the spectrum of such a QD with well‐separated lines. Neutral exciton (X) and biexciton (XX) lines are marked and show a biexciton binding energy above 4 meV. The lines are identified using power‐dependent exponent analysis, polarization‐dependent FSS analysis, and decay‐time‐based comparison, indicating the strong‐confinement regime. Moreover, the polarization‐dependent PL shown in Figure 3c,d reveals the same FSS value of ≈70 μeV for both lines with the expected antiphase dependence. Although the obtained FSS values are comparable to or lower than the spectral resolution ( ∼ 70 μeV), the applied method, based on fitting the excitonic line positions as a function of polarization, including antiphase polarization‐dependent oscillations for orthogonal linear polarizations of the split excitonic states, enables the characterization of splitting values several times smaller than this resolution [64, 65]. Supporting Information S1: Figure S6 shows the results of power‐dependent measurements showing power‐law intensity scaling with an exponent of ≈0.8 for the X line and 1.4 for the XX line. Although the ratio of around 1.8 is close to 2, as expected in the strong‐confinement regime, the experimental exponent values are lower than the expected values of 1 for X and 2 for XX. Those lower values, especially for the XX line, could be related to several effects: (i) significant contribution of the charge exciton (five‐level system instead of three‐level) shaping the power‐dependent excitonic complexes emission intensity combined with the impact of the confinement potential dictating the ratio of the exciton and biexciton decay time [66] and (ii) the impact of the slow feeding processes and the other nonradiative processes limiting population of the system, all affecting emission dynamics. Figure 3e shows decay times of ≈1.06 ns (≈0.48 ns) for the X (XX) line with a ratio of about 2.2 in agreement with power‐dependent analysis, supporting a strong confinement regime identification. The obtained decay time in the single‐ns range, with an average value of about 1.345 ns, lies within standard values of about 1–2 ns for telecom InP‐substrate‐based and GaAs‐substrate‐based high‐quality QDs [1], suggesting rather limited impact of the nonradiative processes. Typically, a reduced decay time in LDE GaAs‐based QDs [67] is not clearly observed in our case with only a few cases showing decay times close to 1 ns. Most likely, the confinement potential for electrons and holes and the correlated wavefunction overlap, which significantly affects the radiative recombination rate, does not support the faster emission dynamics. Moreover, residual strain, even if significantly limited, still exists in that system (see Figure 2c) affecting the lifetime. The obtained results are consistent with theoretical simulations showing a lifetime of 1.3–1.6 ns that is weakly correlated with the dome size (see Figure 2l). The limited wavefunction overlap for electron and hole (see probability density across QD presented in the Figure 2e,f) supports this interpretation where likely the residual strain is mostly contributing. Additionally, the residual asymmetry could further reduce the overlap of the mentioned wavefunctions, thereby increasing the lifetime.

The presented results with higher XX binding and indications of confinement closer to the strong‐confinement regime for this QD, compared to our theoretical predictions, suggest a reduced dome size. Thus, we infer that such less typical QDs are associated with a smaller amount of active material accumulated within and above a nanohole, that is, reduced or vanishing dome size. Although such a difference in morphology significantly affects the confinement potential and binding energies, the exciton decay time stays at a similar level in good agreement with theoretical predictions. The increased separation of the electron and hole states for a reduced QD dome size can also be correlated with higher quenching energies observed for other QDs showing a similar larger separation of the excitonic lines (see Supporting Information S1: Figure S7).

Atypical QDs also reveal nonzero FSS, suggesting an increased anisotropy of the confinement potential. However, these higher FSS values (<70 μeV) presented in Figure 3d and 40 μeV shown in Supporting Information S1: Figure S8 still represent an improvement compared with standard Stranski–Krastanov InP‐substrate‐based QDs with >100 μeV FSS [68]. Compared to more developed droplet epitaxy of InP‐substrate‐based QDs (FSS below ∼ 30 μeV) [9, 68], ripening epitaxy of InP‐substrate‐based QDs (FSS below ∼ 20 μeV) [69, 70], and metamorphic buffer‐layer GaAs‐substrate‐based QDs (FSS below ∼ 5 μeV) [8, 10, 71], the results obtained here still indicate only a limited reduction in FSS. These moderate FSS values are most likely upward‐biased for the studied system due to our limited spectral resolution and moderate linewidths above 100 μeV, which prevent the identification of neutral excitonic complexes with FSS lower than 10–20 μeV, a few times below setup resolution [64, 65]. Observation of such FSS despite the high symmetry of the nanoholes can be understood based on the very low strain in LDE QDs, which results in close‐to‐degenerate heavy and light hole sub‐bands and strongly enhances any band mixing effects [72, 73]. Heavy–light hole mixing in the presence of electron‐hole exchange interaction, in turn, is one of the major contributions to FSS [74, 75]. This points to strain tuning based on creating light‐hole admixtures with a specific phase that cancels the original effect, as a promising route for reducing FSS.

To further verify the potential of the investigated QDs for single‐photon emission, in Figure 3g we show the autocorrelation function obtained using a CW 650 nm excitation laser, demonstrating single‐photon emission with clear antibunching for an exemplary, less typical QD. In this case, a larger line separation allows for applying higher excitation power and obtaining an improved count rate, further enhanced by the high intensity of the used charged exciton line compared to other lines. This allowed for autocorrelation measurements under pulsed excitation at an 80 MHz repetition rate with the results shown in Figure 3f. Both experiments revealed as‐measured $g^{\left(2\right)}\left(0\right)<0.07$, indicating the potential of QDs as single‐photon sources, especially when integrated in photonic structures. The fitting of the autocorrelation data showed $g^{\left(2\right)}\left(0\right)=0.093\;\pm 0.054$ value for CW and $g^{\left(2\right)}\left(0\right)=0.16\pm 0.18$ for pulsed excitation. Moreover, the pulsed autocorrelation data are affected by the observed reexcitation process, which shows additional photons after the first emission, thereby increasing the signal of $g^{\left(2\right)}\left(\tau \right)$ close to zero delay. The exact fitting of $g^{\left(2\right)}\left(0\right)$ is hindered by the limited resolution of the obtained data. Additionally, the observed background in the autocorrelation measurements arises from consecutive peaks that contribute coincidences over the delay‐time interval due to an additional slow‐decay time component of around 5.8 ns for that QD (see Supplementary Supporting Information S1: Figure S8). However, based on the fit to the autocorrelation data, we also find that a portion of the background originates from weak background emission, thereby degrading the $g^{\left(2\right)}\left(0\right)$ value. Although employing more advanced quasi‐resonant or less‐detuned excitation schemes could mitigate the impact of both background emission and slow feeding processes by directly populating the investigated QD, implementing such methods lies beyond the scope of this manuscript.

Spectra obtained for QDs exhibiting small energy separations (presented in Supporting Information S1: Figure S5) are consistent with theoretical predictions, indicating low binding energies of excitonic complexes particularly for QDs incorporating the full‐dome structure. The two dominant lines that exchange intensity with excitation power are attributed to the X‐XX pair with a splitting of ≈0.41 meV, whereas the third line corresponds to a charged exciton separated by ≈1.7 meV in agreement with simulations. Measured decay times range from 0.94 to 2.11 ns (average ≈1.345 ns) comparable to theoretical values, supporting negligible nonradiative losses. A weak, slow component likely arises from delayed carrier feeding with no clear evidence of additional degradation mechanisms. Arrhenius analysis reveals a low activation energy of ≈4.6 meV (see Supporting Information S1: Figure S5f) consistent with thermal population of excited hole states rather than carrier escape. Overall, the good agreement with theory provides insight into the observed behavior and suggests routes toward improved thermal stability, for example, by optimizing the filling step to increase carrier level spacing. Statistical analysis yields DOLP ≈9%, pointing to high in‐plane symmetry and low valence‐band mixing, in contrast to typical Stranski–Krastanov InP‐based QDs [68].

## 5 Conclusions

We have demonstrated LDE‐assisted epitaxial growth of low‐density ${\text{In}}_{0.53}{\text{Ga}}_{0.47}$As QDs embedded in an ${\text{In}}_{0.52}{\text{Al}}_{0.48}\text{As}$ matrix lattice‐matched to InP, emitting in the telecom C‐band with single‐photon purity ∼93%. Structural and morphological characterization confirmed a two‐section QD geometry composed of a symmetric cone‐like structure based on a filled nanohole and a slightly asymmetric dome. Theoretical modeling showed that with decreasing dome size, the confinement evolves from an intermediate toward a stronger confinement regime for the exciton, whereas the radiative lifetimes of X and XX change only weakly, underlining the resilience of the basic optical QD properties to such morphological variations.

Optical experiments were performed for QDs with X‐XX binding energies comparable to the linewidth consistent with theoretical predictions and for QDs with a larger X‐XX binding energy of 4 meV, which is more suitable for FSS estimation. The FSS was found to be on the order of tens of μeV, indicating that further optimization of growth conditions combined with post‐growth electrical or mechanical tuning will be required to achieve a platform for high‐quality polarization‐entangled photon sources operating at telecom wavelengths based on the ${\text{In}}_{0.53}{\text{Ga}}_{0.47}\text{As}$/${\mathrm{In}}_{0.52}{\text{Al}}_{0.48}\text{As}$ system.

## 6 Methods

### 6.1 Epitaxy

The samples were grown using the TurboDisc metal‐organic vapor phase epitaxy (MOVPE) reactor using arsine (${\text{AsH}}_{3}$), phosphine (${\text{PH}}_{3}$), trimethylaluminum (TMAl), trimethylgallium (TMGa), and trimethylindium (TMIn) precursors with $H_{2}$ serving as a carrier gas. On top of the InP (100)‐oriented substrate, a 500 nm InP buffer layer was grown, followed by a 50 nm lattice‐matched ${\text{In}}_{0.52}{\text{Al}}_{0.48}\text{As}$ barrier layer. The sample was then cooled from the growth temperature of 610°C to 400°C, and In droplets were deposited on the surface and annealed for 10 s in an ${\text{AsH}}_{3}$ background. Under such conditions, the LDE takes place [41, 44, 50, 51, 52, 57, 76]. After etching, the sample was reheated to the growth temperature of 610°C, and the nanoholes were filled with QD material, which was equivalent to a 2 nm layer of ${\text{In}}_{0.53}{\text{Ga}}_{0.47}\text{As}$, lattice‐matched to the ${\text{In}}_{0.52}{\text{Al}}_{0.48}\text{As}$ layer below and to the InP substrate to maintain strain‐free conditions. Finally, QDs were capped with 50 nm of ${\text{In}}_{0.52}{\text{Al}}_{0.48}\text{As}$.

### 6.2 Microscopy

Transmission electron microscopy (TEM) imaging was performed using a Thermo Fisher Spectra Ultra (S)TEM instrument equipped with a probe aberration corrector and operated at an acceleration voltage of 300 kV. The concentration of different elements was determined from X‐ray energy‐dispersive spectroscopy (EDS) data collected using the ultra EDS system. Dark‐field high‐angle annular dark‐field Scanning TEM (HAADF STEM) images were acquired with a probe convergence semi‐angle of 30 mrad and a collection semi‐angle of approximately 50 mrad. The HAADF STEM and EDS data were processed and analyzed using Velox from Thermo Fisher Scientific (Version 3.14.0) and DigitalMicrograph from Gatan (Version 3.60.4435.0) software.

Atomic force microscope (AFM) imaging was performed in PeakForce Tapping mode using the Bruker AFM Dimension Icon‐Pt microscope equipped with the Tap150Al‐G Soft Tapping Mode AFM Probe by BudgetSensors.

### 6.3 Optical Experiments

The samples were optically characterized using a custom‐built micro‐photoluminescence (μPL) setup. To perform low‐temperature characterization, the sample was mounted inside a closed‐cycle cryostat (attoDRY800xs, Attocube) operating at a base temperature of ∼4 K, equipped with piezoelectric nanopositioners and a high‐numerical‐aperture telecom microscope objective (60×, NA = 0.8, LT‐APO/Telecom, Attocube) integrated within the cryostat chamber. Excitation of the QDs was performed using semiconductor diode lasers (PicoQuant) at 650 nm (LDH‐D‐C‐650), operated in continuous‐wave (CW) or pulsed modes, with pulses shorter than 100 ps and adjustable repetition rates ranging from 2.5 to 80 MHz. Spectral analysis of the QDs emission was investigated using a spectrometer based on a 0.328 m focal‐length monochromator (Kymera 328i, Andor, Oxford Instruments) equipped with interchangeable gratings (150 and 600 lines/mm) combined with a deep‐thermo‐electrically cooled (In,Ga)As linear array detector (iDus 1.7 μm, Andor, Oxford Instruments), providing a maximum spectral resolution of ∼70 μeV. Polarization‐resolved μPL measurements were performed using a rotating half‐wave plate followed by a linear polarizer placed in the detection path. Time‐correlated single‐photon‐counting mode was employed for time‐resolved μPL characterization and single‐photon statistics measurements. Detection was carried out using superconducting nanowire single‐photon detectors (SNSPDs; ID281, ID Quantique) connected to a high‐resolution single‐photon counting module (Time Tagger Ultra, Swabian Instruments). To verify single‐photon emission, a Hanbury Brown and Twiss (HBT) configuration was used employing a 50:50 telecom fiber beamsplitter.

### 6.4 Theoretical Modeling

To model the QD, we represent the material distribution within a computational box on a regular Cartesian mesh of points. Then, we determine the spatial distribution of the strain tensor $\varepsilon_{ij}\left(r\right)=\left(\partial u_{i}/\partial x_{j}+\partial u_{j}/\partial x_{i}\right)/2+\delta_{ij}\left[a_{\text{ref}}-a\left(r\right)\right]/a\left(r\right)$ with u the displacement field and $a_{\text{ref}}$ the reference (substrate) lattice constant by minimizing the elastic energy of the system $(1/2)\int d^{3}r\sum_{\mathrm{ijkl}}C_{\mathrm{ijkl}}\left(r\right)\varepsilon_{ij}\left(r\right)\varepsilon_{kl}\left(r\right)$ within the framework of continuum elasticity theory where $C_{\mathrm{ijkl}}\left(r\right)$ are the position‐dependent (due to composition variations) elastic stiffness constants of the material. Although the system is weakly strained, we calculate the shear‐strain‐induced piezoelectric polarization P(r) up to second‐order terms in the strain tensor elements, $P_{i}=\sum_{j}e_{ij}\varepsilon_{j}+\frac{1}{2}\sum_{jk}B_{\mathrm{ijk}}\varepsilon_{j}\varepsilon_{k}$ where Voigt notation is used for the strain components, and $e_{ij}$ and $B_{\mathrm{ijk}}$ denote the first‐ and second‐order piezoelectric tensors, respectively. P(r) gives rise to an inhomogeneous electrostatic potential φ(r) obtained by solving Poisson's equation, $\nabla \;\cdot \;\left[\varepsilon_{0}\varepsilon_{r}\left(r\right)\nabla \varphi \left(r\right)\right]=\nabla \;\cdot \;P\left(r\right)$.

The composition profile, strain, and piezoelectric potential are then used to calculate electron and hole eigenstates employing the custom implementation [77, 78] of the 8‐band k⋅p method in the envelope function approximation [79], also including spin‐orbit effects. The explicit Hamiltonian used can be found in Ref. [78], whereas the material parameters used are listed in Ref. [60] and were collected from Refs. [80, 81, 82, 83]. The diagonalization of the k⋅p Hamiltonian yields the eigenstates as eight‐component pseudospinors $\psi \left(r\right)=\sum_{\mu }\phi_{\mu }\left(r\right)|\mu \rangle$ of envelope functions $\phi_{\mu }\left(r\right)$ for the considered bands (conduction, heavy, light, and split‐off hole bands) labeled by μ. Hole states are calculated by applying the time‐reversal operator to the valence‐band electron states.

Further, we construct an electron‐hole configuration (product) basis using 48 electron and 48 hole states in which we calculate the exciton eigenstates, $|X_{n}\rangle =\sum_{ij}c_{ij}^{\left(n\right)}|e_{i}\rangle \;⊗\;|h_{j}\rangle$, with complex coefficients $c_{ij}^{\left(n\right)}$ using the configuration interaction approach [84]. For this, we include the direct Coulomb and identical‐particle exchange interactions as well as a phenomenological electron‐hole exchange interaction. Finally, we evaluate within the dipole approximation the optical transition dipole moments $d_{n}=e/\left(m_{0}\omega_{n}\right)\;\langle \emptyset |p|X_{n}\rangle$ with |∅〉 denoting the empty QD state, $p=m_{0}\hbar^{-1}\;\nabla_{k}H\left(k\right)$, and the resulting recombination times $\tau_{n}=3\pi \varepsilon_{0}\hbar c^{3}/\left(n_{r}\omega_{n}^{3}|d_{n}{|}^{2}\right)$ where $\omega_{n}=E_{n}/\hbar$ is the transition angular frequency, and $n_{r}$ is the refractive index of the surrounding medium. States of other carrier complexes and their optical transition dipoles are calculated similarly, for example, biexciton states are calculated in the four‐particle configuration basis as $|{\text{XX}}_{m}\rangle =\sum_{\mathrm{ijkl}}c_{\mathrm{ijkl}}^{\left(m\right)}|e_{i}\rangle \;⊗\;|e_{j}\rangle \;⊗\;|h_{k}\rangle \;⊗\;|h_{l}\rangle$ with transition dipole moments $d_{nm}=e\langle X_{n}|p|{\text{XX}}_{m}\rangle /\left(m_{0}\omega_{mn}\right)$ where $\omega_{mn}=\left(E_{m}-E_{n}\right)/\hbar$.

## Author Contributions

**Karolina E. Połczyńska:** methodology, investigation, formal analysis, visualization, writing – original draft, writing – review and editing, conceptualization. **Paweł Wyborski:** investigation, formal analysis, writing – review and editing. **Michał Gawełczyk:** investigation, visualization, formal analysis, writing – review and editing. **Shima Kadkhodazadeh:** investigation, formal analysis. **Battulga Munkhbat:** investigation. **Stefano Sanguinetti:** conceptualization. **Elizaveta Semenova:** conceptualization, supervision, writing – review and editing, resources, funding acquisition.

## Conflicts of Interest

The authors declare no conflicts of interest.

## Supporting information


Supporting Information S1

## Data Availability

The data that support the findings of this study are available from the corresponding author upon reasonable request.
