# Supplementary material for: Local Droplet Etching‐Assisted Quantum Dot Epitaxy for Telecom C‐Band Quantum Light Emitters
Source: Nanophotonics. 2026 Oct 16;15(20):e70310. doi: 10.1002/nap2.70310 (PMC13673233; doi:10.1002/nap2.70310)
Supplement: Supplementary file 1 [file NAP2-15-e70310-s001.pdf]

Local droplet etching–assisted quantum dot epitaxy for telecom C-band quantum light emitters - Supplementary Information

Karolina E. Połczyńska Paweł Wyborski Michał Gawełczyk Shima Kadkhodazadeh Battulga Munkhbat Stefano Sanguinetti Elizaveta Semenova*

Karolina E. Połczyńska, Paweł Wyborski, Battulga Munkhbat, Elizaveta Semenova
DTU Electro, Technical University of Denmark, Address: Ørsteds Plads 343, 2800 Kongens Lyngby, Denmark
Email Address: karpo@dtu.dk
Shima Kadkhodazadeh
DTU Nanolab, Technical University of Denmark, Address: Fysikvej 307, 2800 Kongens Lyngby, Denmark
Karolina E. Połczyńska, Shima Kadkhodazadeh, Elizaveta Semenova
NanoPhoton-Center for Nanophotonics, Technical University of Denmark, Address: Ørsteds Plads 343, 2800 Kongens Lyngby, Denmark
Michał Gawełczyk
Institute of Theoretical Physics, Wrocław University of Science and Technology Address: Wybrzeże Wyspiańskiego 27, 50-370 Wrocław, Poland
Stefano Sanguinetti
Department of Materials Science, University of Milano-Bicocca Address: Via Cozzi 55, 20125 Milano, Italy

Keywords: *local droplet etching epitaxy, quantum dots, telecom C-band*

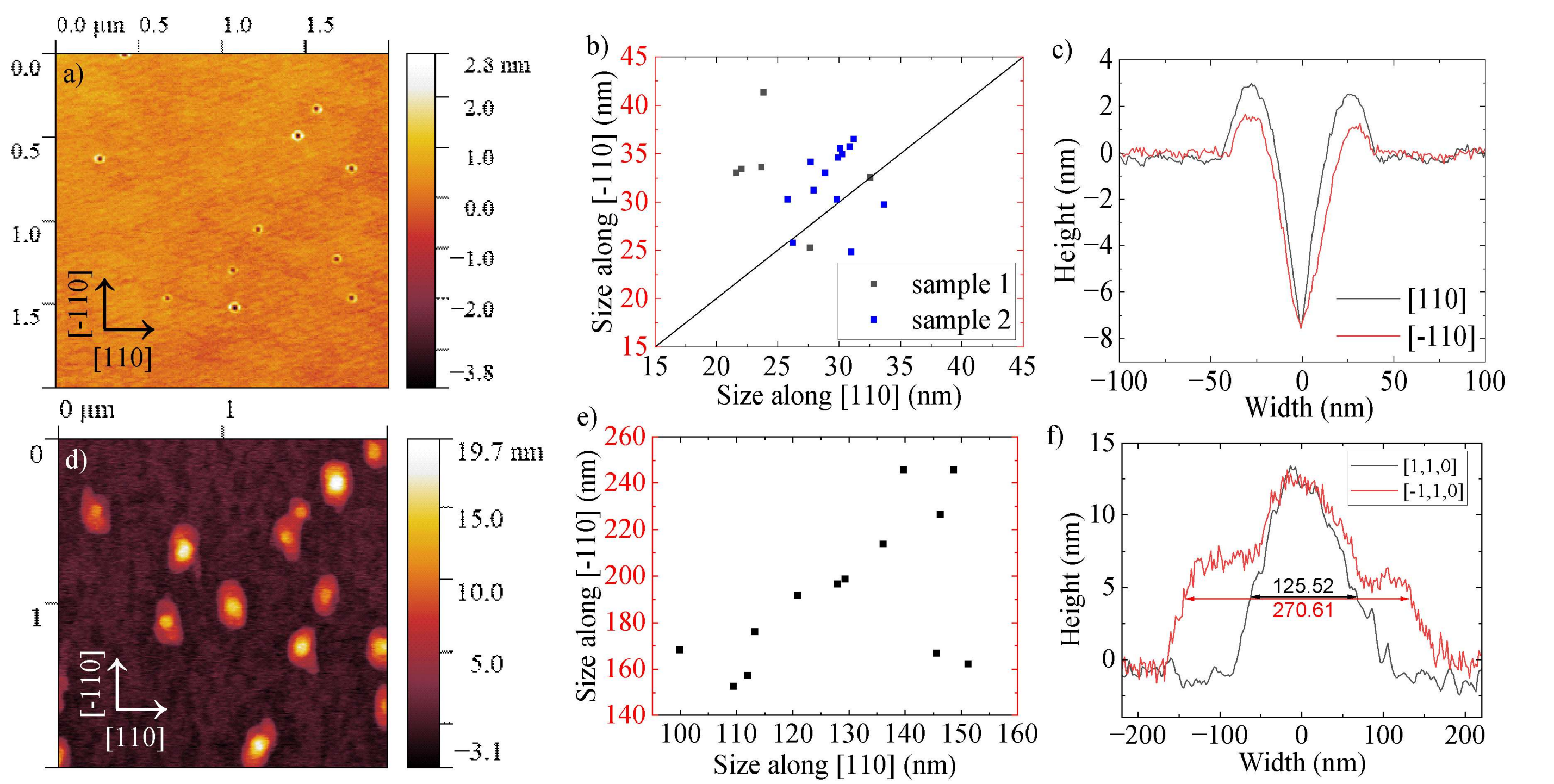

Figure S1: Nanoholes etched in the $In_{0.52}Al_{0.48}As$ layer by In droplets imaged by AFM: a) picture of the surface with etched nanoholes, b) width of nanoholes measured at the surface level along different crystallographic directions, c) cross-sections along the different crystallographic directions of the nanohole from the figure 1e from the main article. Uncapped QDs: LDE nanoholes filled with 2 nm of $In_{0.53}Ga_{0.47}As$ imaged by AFM: d) picture of the surface with QDs, e) width of the QDs measured at the surface level along different crystallographic directions, f) cross-sections along the different crystallographic directions of the QD from the picture in Fig 1f from the main article

The LDE process resulted in nanoholes in the $In_{0.52}Al_{0.48}As$ layer with a surface density of $\sim 10^9$ cm^{-2}, as presented in Fig. S1. AFM analysis revealed mean diameters of 30.5 nm along [110] and 34.8 nm along [−110], corresponding to a near-cylindrical symmetry with an aspect ratio of 1.14, and an average depth of approximately

Growth step	Material grown	Temperature (K)	Time (s)	Precursor partial pressures (mbar)	III/V ratio
Buffer	InP	610	610	In 1.183; P 0.676	0.428
Bottom barrier	InAlAs	610	70.3	In 1.85; Al 0.070; As 0.584	0.348
Cooling down	-	400	453	As 0.004	0.445
Waiting	-	400	60	-	0.463
In droplets	In	400	8.35	In 0.370	0.6
Waiting	-	400	10	-	0.463
Heating up	-	610	462	As 0.004	0.445
Nanohole filling (QDs)	InGaAs	610	2.5	In 0.852; Ga 0.037; As 0.585	0,339
Waiting	-	610	5	As 0.585	0.343
Cap layer	InAlAs	610	70.3	In 1.186; Al 0.070; As 0.585	0.348

Table 1: Growth conditions for each growth step, including temperature, duration, precursor partial pressure, and III/V ratio.

7.5 nm. The nanoholes exhibit a slight elongation and anisotropy consistent with the LDE mechanism. Deposition of 2 nm of $In_{0.53}Ga_{0.47}As$ leads to nanohole overfilling and the formation of quantum dots with an average height of 15 nm and lateral elongation of 30% along $[-110]$. The resulting QD density closely matches the initial nanohole density, confirming deterministic nucleation. Growth conditions during each of the growth steps are presented in Table 1.

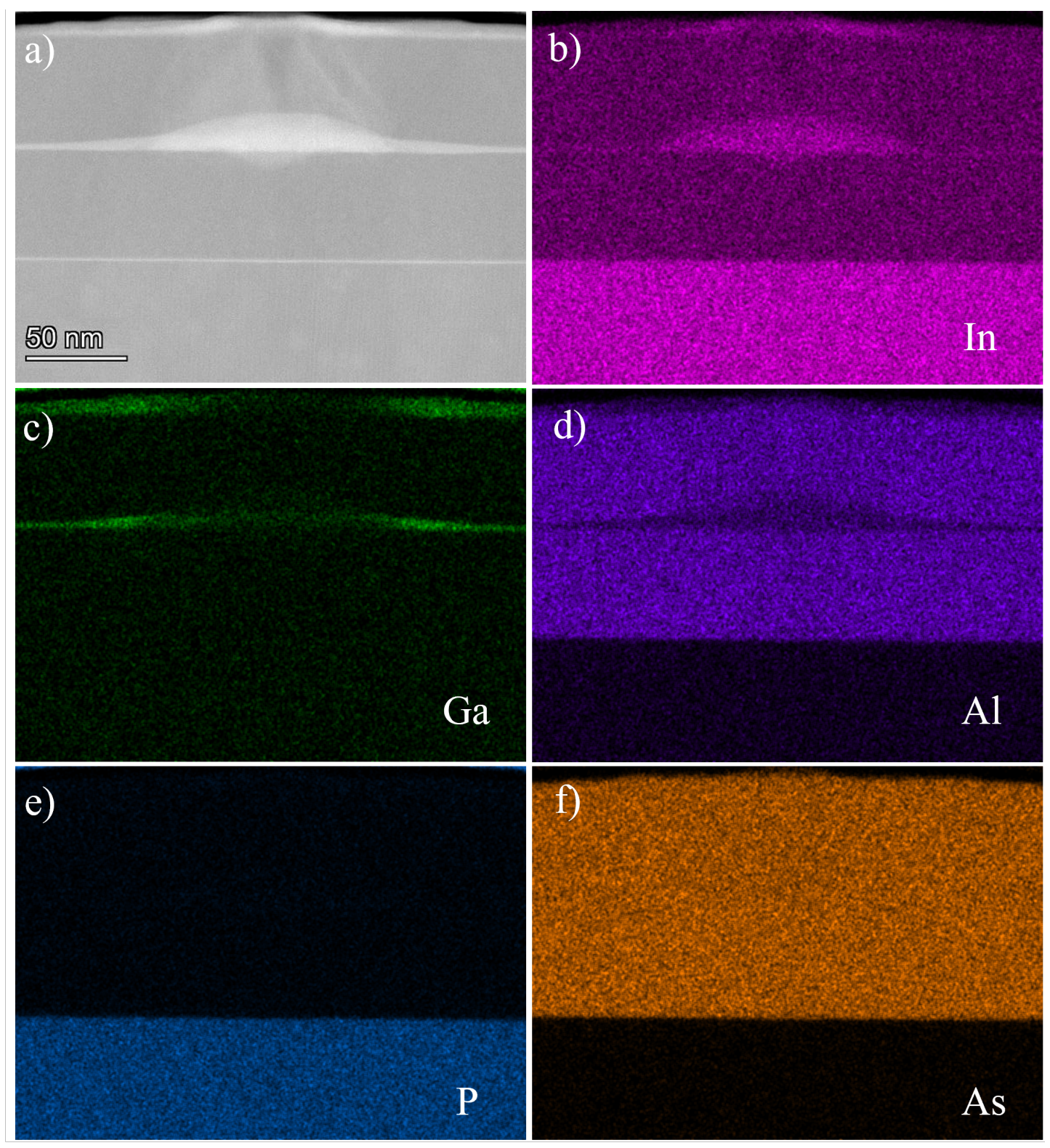

Figure S2: Chemical composition of the QD and its surrounding area. a) TEM picture of the QD and its 2D elemental composition maps by EDS of b) In (magenta), c) Ga (green), d) Al (purple), e) P (blue), and f) As (orange), respectively.

Fig. S2 presents a graphical representation of the EDS signal obtained from the sample. The elements of interest include indium, gallium, aluminum, phosphorus, and arsenic. A detailed analysis of the EDS data allowed us to determine the elemental composition of the quantum dot ($In_{0.88}Ga_{0.12}As$), nanoring ($In_{0.86}Ga_{0.1}Al_{0.04}As$), and the barrier ($In_{0.52}Al_{0.48}As$).

The macro-PL spectrum from Fig. S3 shows emission from the InP substrate at 1141 meV (1086 nm), barrier-related emission between 1058–1184 meV (1047-–1172 nm), and single QD emission in the range of 752–950 meV (1305-–1648 nm). Micro-PL measurements further confirm emission from individual quantum dots at different positions across the sample.

Detailed optical characterization should not only allow investigation of QD properties, but also comparison

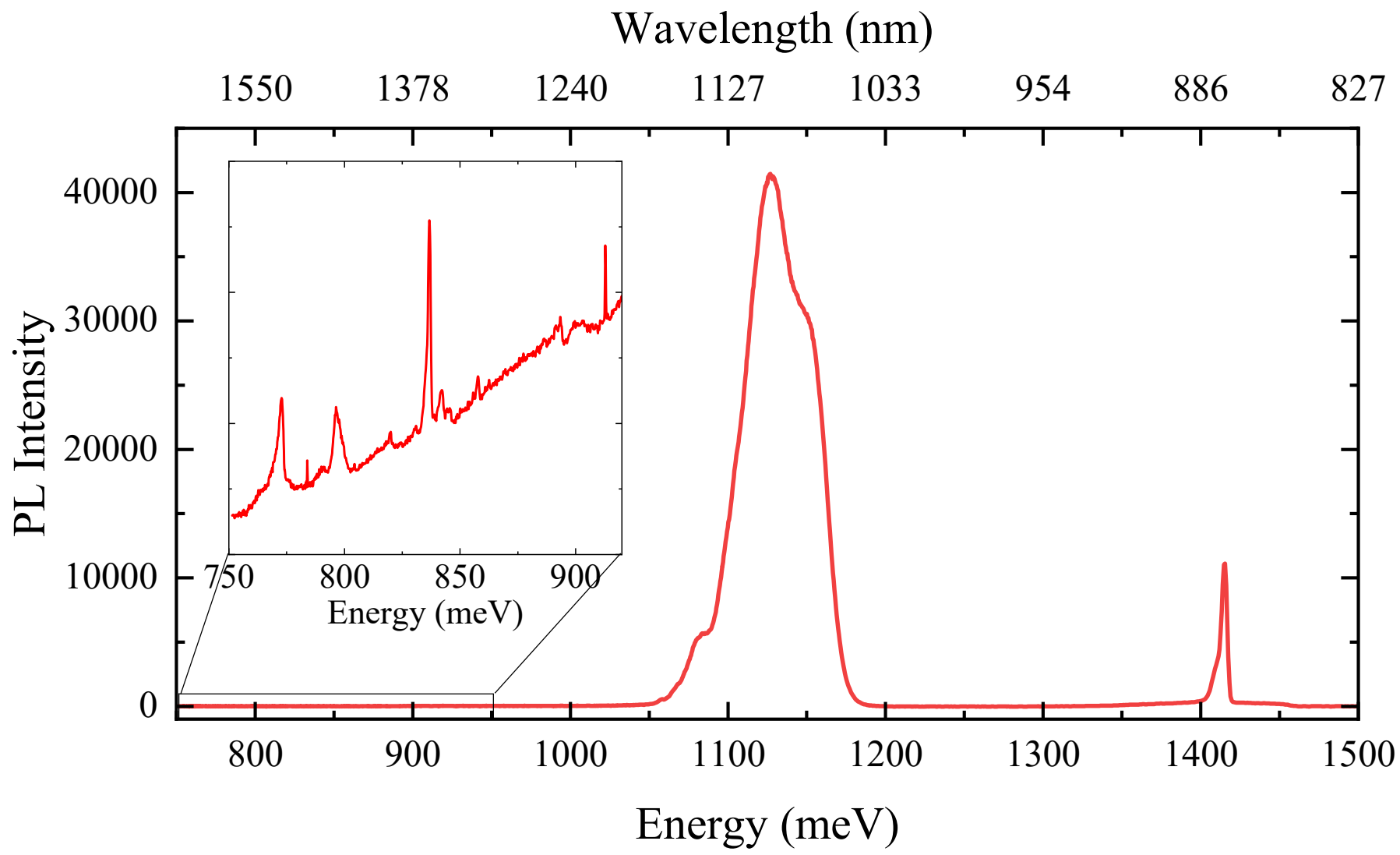

Figure S3: Photoluminescence signal of the sample measured at 5 K under 650 nm CW excitation. Macro-PL from the InP substrate at 1141 meV (1086 nm), emission related to the barrier between 1058–1184 meV (1047–1172 nm) and single-QD emission around 752–950 meV (1305–1648 nm). Insert: macro-PL from the QDs spectral region.

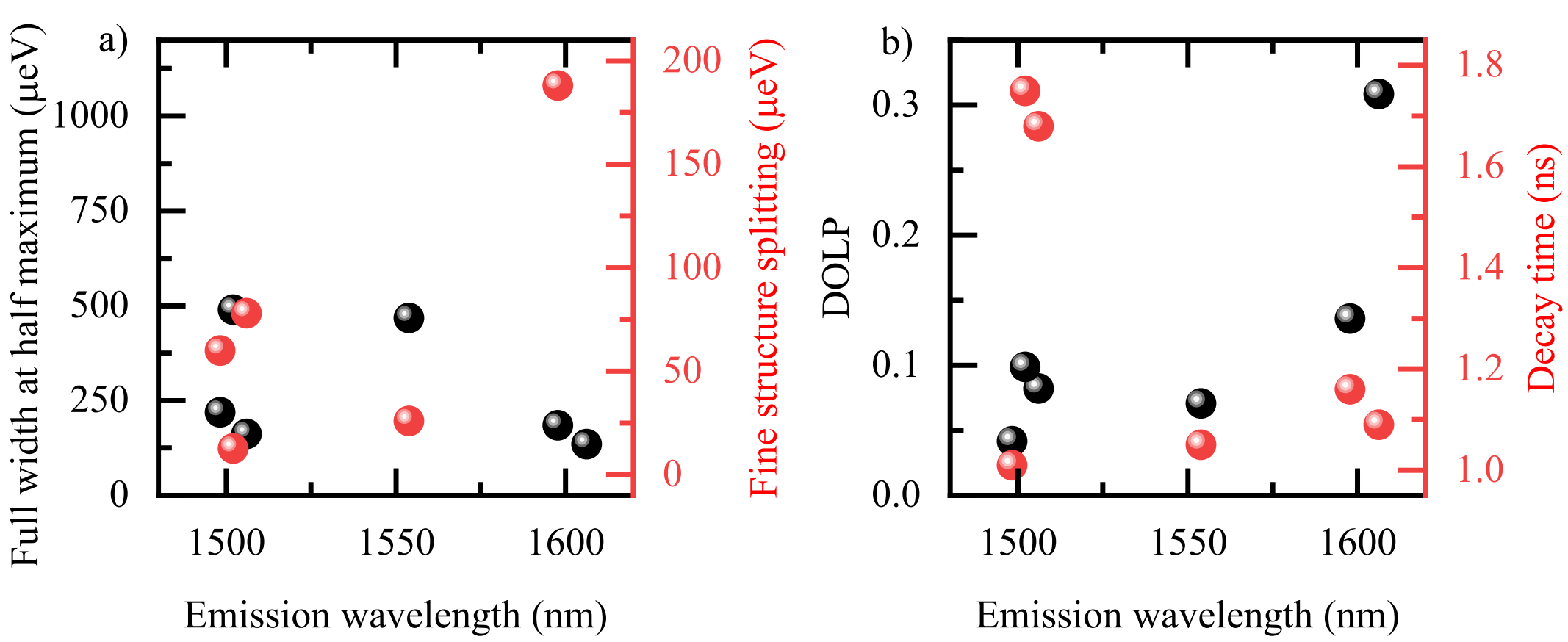

Figure S4: Optical properties of the representative ensemble of investigated quantum dots. a) Fine-structure splitting (FSS) and emission linewidth (FWHM) as a function of emission wavelength. b) Degree of linear polarization (DOLP) and exciton lifetime as a function of emission wavelength. The quantum dots analyzed in detail in the main text are included in this dataset. Mean values based on those data: FWHM = 202 μeV, FSS = 60 μeV, DOLP = 0.0906, decay time 1.345 ns.

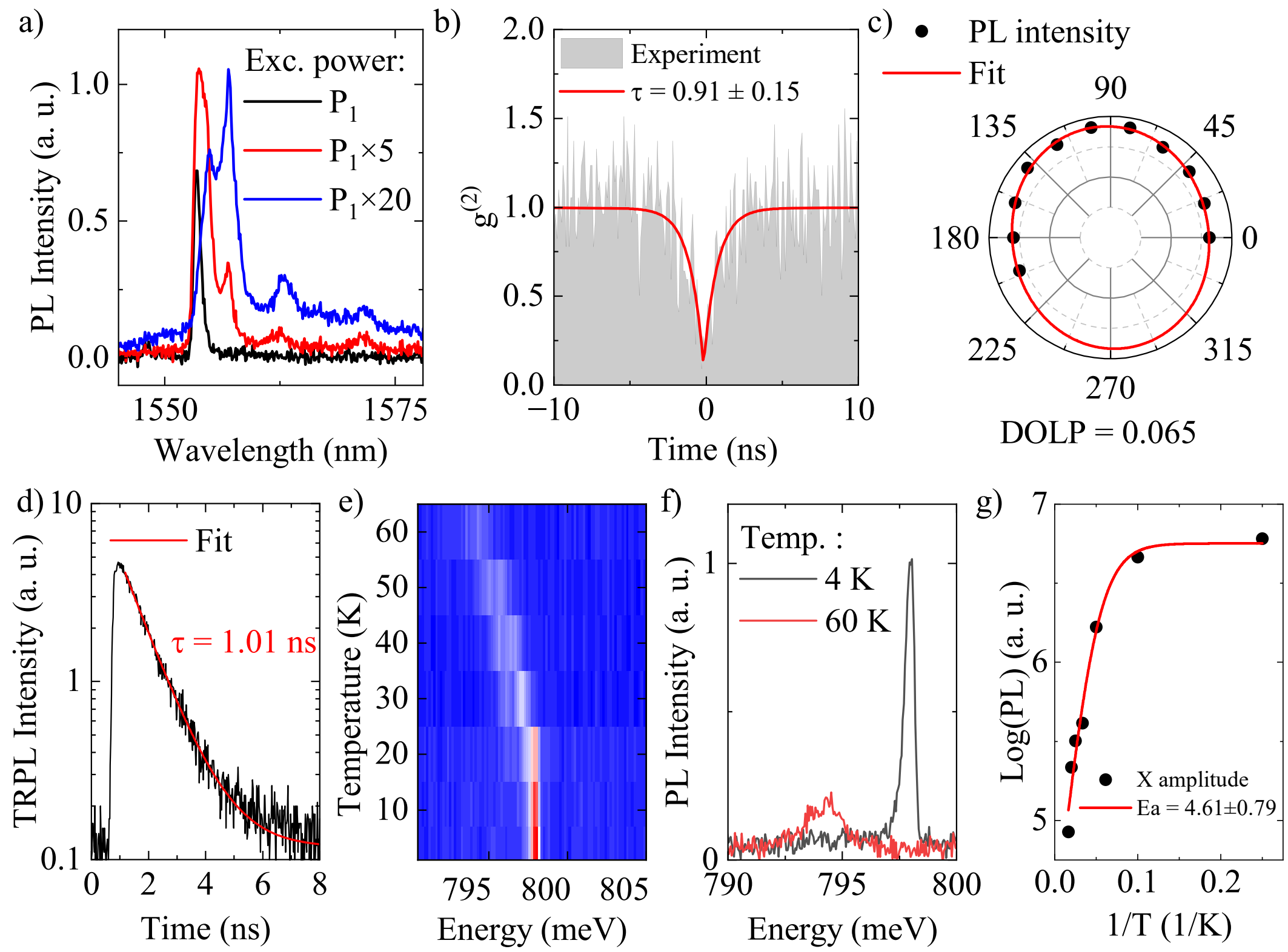

Figure S5: Optical properties of the single QD. a) μPL spectrum of an individual QD measured for different power of excitation. b) g(2) measured with CW excitation of 660 nm. Experimental $g^{(2)}(0) = 0.09$, fit: $g^{(2)}(0) = 0.12 \pm 0.10$. c) μPL intensity as a function of the angle of polarization. DOLP $= 0.065 \pm 0.007$. d) Time-resolved photoluminescence, decay time from the fit $\tau_{1X} = 1.01 \pm 0.005$ ns. e) μPL measured for different values of the temperature. f) Comparison of the optical signal from the QD measured at 4 K and 60 K. g) Temperature dependence of the X line μPL intensity with the Arrhenius fit line. Activation energy from the fitting: $E_a = 4.61 \pm 0.79$ meV.

with our theoretical predictions. Fig. S3 presents the QD emission at the center of the C-band, representing the near-median of the distribution, thus expected to come from a typical QD well suited for comparison with the theoretical predictions. Low-power excitation data reveal a narrow QD line with FWHM $\approx$ 255 μeV (spectral resolution of $\approx$70 μeV), suggesting still significant spectral diffusion affecting QD linewidth. Statistical analysis of 6 QDs revealed average line broadening around 200 μeV in agreement with the presented spectrum. The two higher-power excitation spectra in Fig. S3a show additional broadening of the main line. However, this broadening can be attributed to the presence of other lines close to the main line, as predicted theoretically for the excited state of the positive trion, which are expected to increase with the excitation power. The low separation between lines hinders particular verification of those lines' properties, specifically polarization-dependent and power-dependent properties, as well as decay time, making their identification challenging. However, the obtained spectra and their low separation agree with calculation predictions, suggesting low binding energies for other excitonic complexes, especially when the QD shape includes the full size of the dome structure. Based on this agreement, we can assume the two strongest lines that exchange the intensity as the excitation power is increased to be an X-XX pair, with a splitting of $\approx$ 0.41 meV, and the third visible line to be a charged exciton split from X by $\approx$ 1.7 meV, which is consistent with the simulation.

Despite these challenges, we investigated main line polarization anisotropy, emission dynamics, and temperature dependence. Starting from the Hanbury Brown and Twiss configuration, we investigated single-photon emission. Fig. S5b presents the autocorrelation function obtained using CW 650 nm excitation laser, demonstrating single-photon emission with clear antibunching and a measured $g^{(2)}(\tau) < 0.09$. The standard fitting revealed a value of 0.115 ± 0.101. Additionally, time-resolved PL measurements (TRPL) presented in Fig. S5d provide insight into emission dynamics, showing a decay time of 1.01 ± 0.005 ns. Characterization of the main decay time for other QDs revealed values ranging from 0.94 ns to 2.11 ns with an average value $\approx$ 1.41 ns. These observed values are slightly lower than the theoretical prediction (see Fig. 2), consistent with the expected underestimation of the radiative rate in the calculation. These results, compared to a standard range of decay time values for InAs QDs around 1-2 ns [1, 2, 3], suggest good optical quality of the investigated QDs, indicating no significant impact of non-radiative processes reducing QDs' quantum efficiency. The weak and slower decay component is likely connected to a slow channel of carrier feeding to a QD after nonresonant excitation observed for other LDE QDs [4]. We cannot also exclude additional processes of carrier transfer to local charge traps. However, we do not observe other deterioration effects expected in that case.

Polarization-resolved measurements presented in Fig. S5c show rather weak anisotropy with a degree of linear polarization (DOLP) of $6.5 \pm 0.69\%$. Statistical analysis of the investigated QDs reveals DOLP $\approx 5.43\%$. This highly symmetric polarization of QD emission indicates a high in-plane symmetry of the QD confinement potential (and the resulting low light-hole subband admixture to the hole ground state, especially compared with Stranski-Krastanov InP-substrate-based QDs. The fine structure splitting of the neutral excitonic transition also directly results from a reduced in-plane symmetry, and is thus indirectly connected with DOLP. Therefore, low observed DOLP values can indicate a potential for low FSS in these QDs. Direct verification of the FSS for the line in question was challenging due to its low intensity and the overlap with other lines at higher powers. However, we further analyze other QDs, showing FSS$<$ 70 μeV. Together with low DOLP, this suggests the potential of the used epitaxial growth for obtaining low-FSS QDs grown on the InP substrate, in contrast to asymmetric Stranski-Krastanov InP-substrate-based QDs [5].

To assess the suitability of QDs as quantum emitters at elevated temperatures, we also investigate their temperature-dependent emission. Fig. S5e presents the temperature series for the investigated main line, showing a significant reduction in intensity above 10 K, however, with emission sustained up to 60 K. We observe standard redshift and line broadening due to thermal expansion and thermally enhanced carrier-phonon interaction [6, 7, 8]. The most important and unfavorable source of emission intensity reduction is the escape of carriers from the QDs. Here, Arrhenius fitting of the main line intensity as a function of temperature shows a low characteristic energy of the emission quenching of $\approx$ 4.6 meV (see Fig. S5g). Such a low energy cannot be associated with carrier escape from a hundreds-of-meV-deep trap. Comparing this value with theoretical predictions of the energy level separations (see Fig. 2i) suggests that the reduced intensity of the main transition is due to the promotion of the hole to excited levels within a QD. While such a process is not as critical as carrier escape, current QDs require cryogenic temperatures to operate as a single- and indistinguishable-photon source. The good agreement with

theoretical predictions not only explains the observed behavior but also allows us to define a clear path to more thermally stable QDs based on the same nanohole etching procedure. A refined filling step recipe preventing the accumulation of material in the dome will lead to ≈ 8 (≈ 3) times larger hole (electron) level spacing.

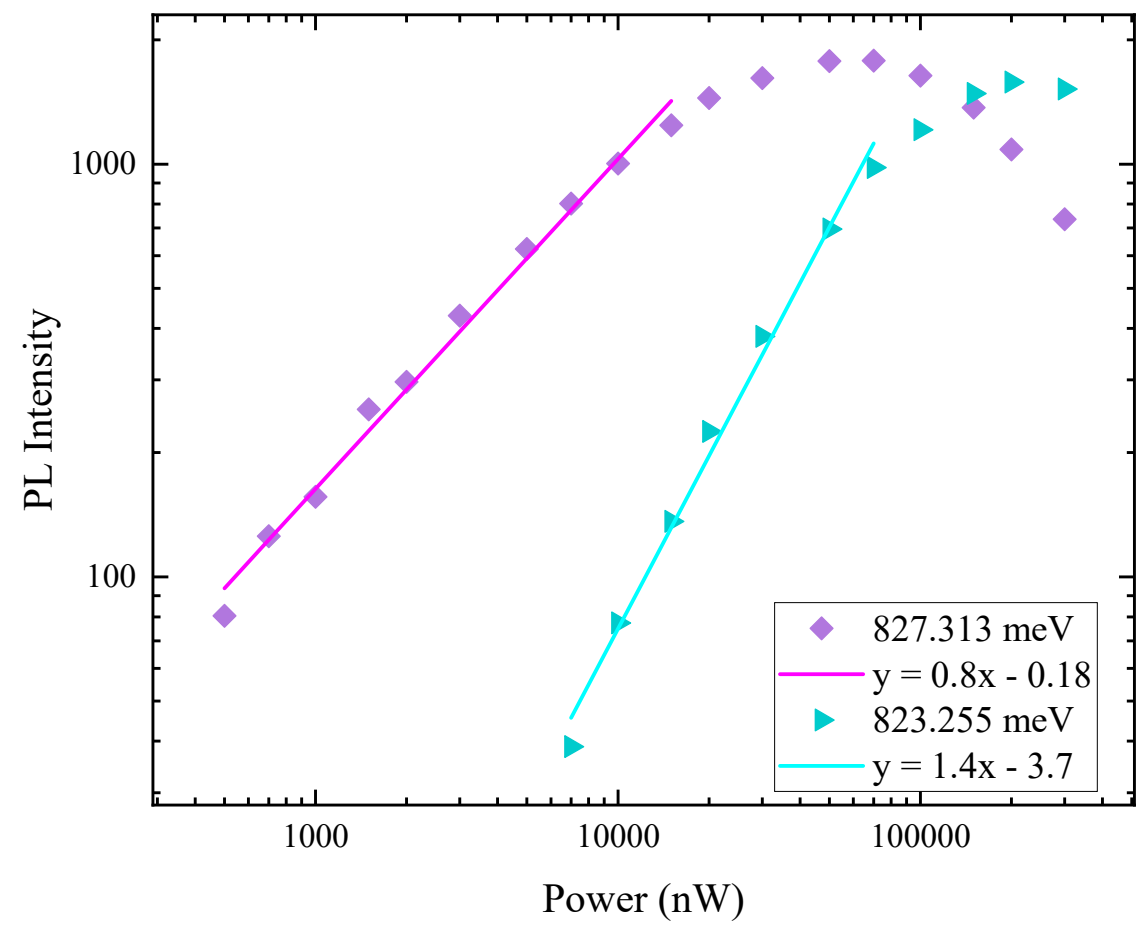

Figure S6: Additional optical properties of the single QD, presented also in Figs. 3b-e from the main article: integrated µPL intensity as a function of excitation power for X and XX with fit lines.

Fig. S6 shows the results of power-dependent measurements of the QD, also presented in Fig. 3 in the main article. The Power-law intensity scales with a 0.8 exponent for the X line and 1.4 for the XX line. The ratio of around 1.8 is close to 2, as expected in the strong confinement regime.

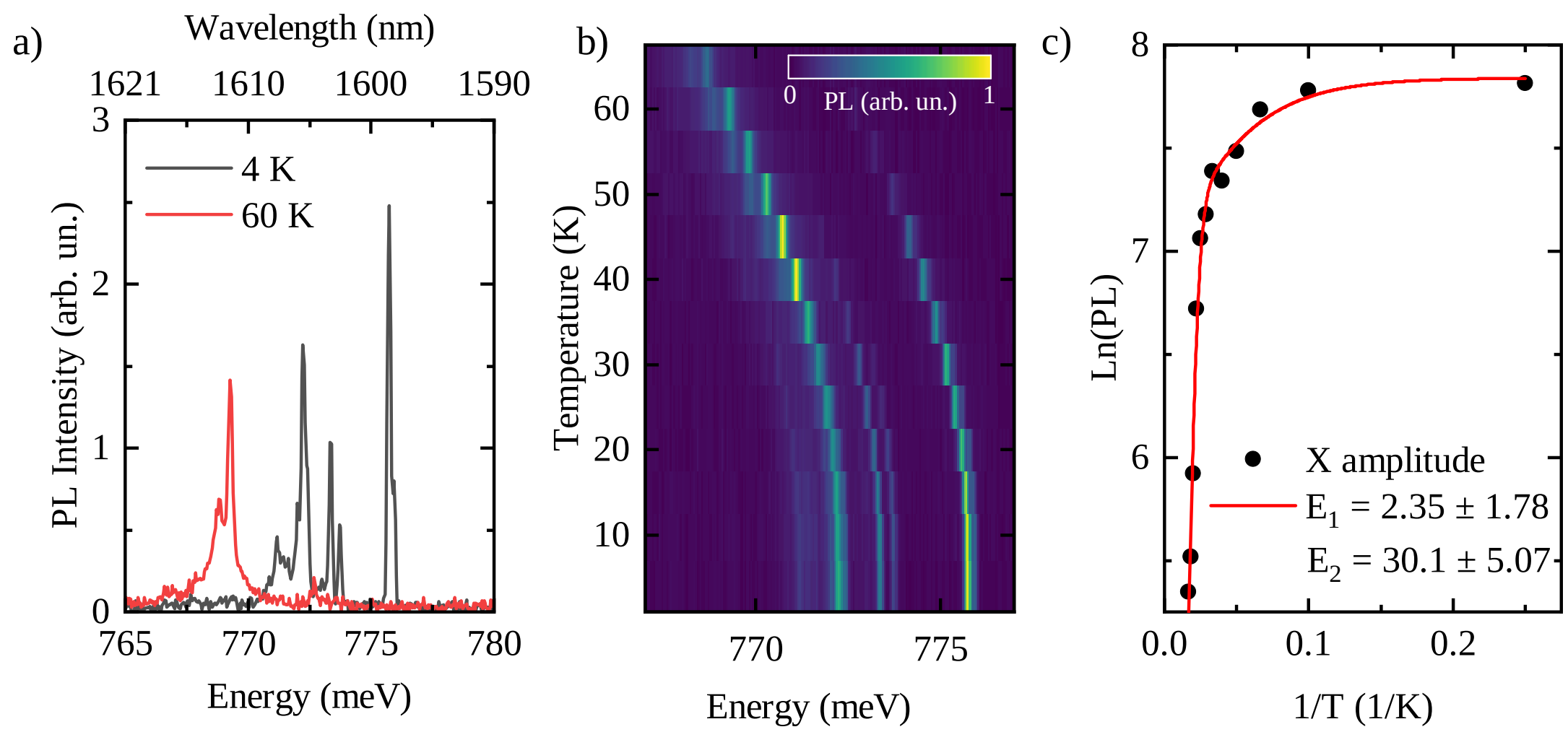

Figure S7: Activation energy measurements: a) Comparison of the optical signal from the QD measured at 4 K and 60 K. b) μPL measured for different values of the temperature. c) Temperature dependence of the X line μPL intensity with the Arrhenius fit line.

An example of a QD with larger separation of the excitonic lines is shown in Fig. S7. The increased separation of the electron and hole states for a reduced QD dome size can also be correlated with higher quenching energies.

References

[1] W. Yang, R. R. Lowe-Webb, H. Lee, P. C. Sercel, *Phys. Rev. B* **1997**, *56* 13314.

[2] J. Johansen, S. Stobbe, I. S. Nikolaev, T. Lund-Hansen, P. T. Kristensen, J. M. Hvam, W. L. Vos, P. Lodahl, *Phys. Rev. B* **2008**, *77* 073303.

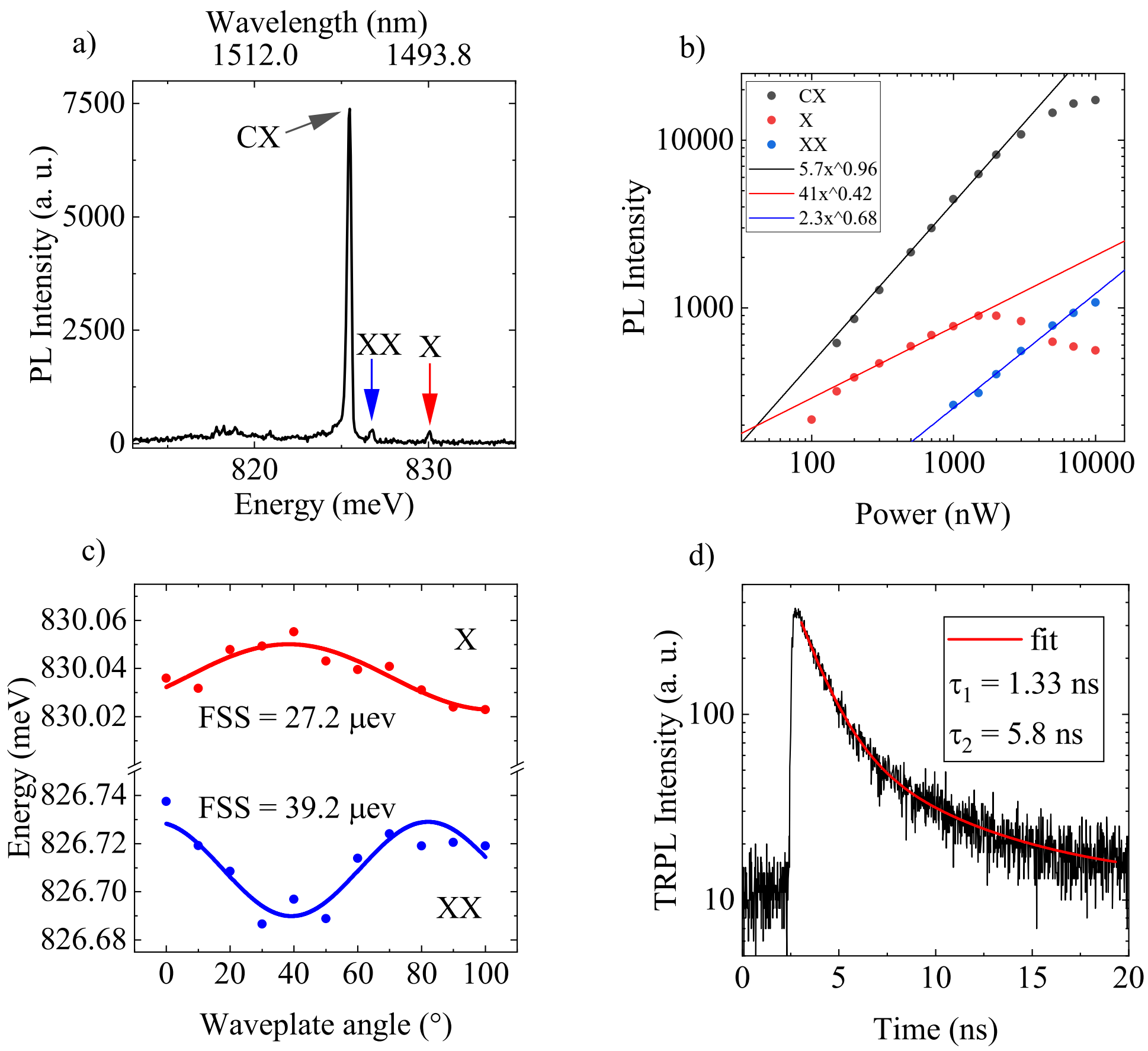

Figure S8: Optical properties of the single QD emitting in the telecom C-band. a) μPL spectrum of the QD with marked X, XX and CX. b) Power dependence of the excitonic line intensities. c) μPL intensity as a function of the angle of polarization for X and XX. FSS obtained from the fitting to X FSS $= 27.2 \pm 2.6 \mu$eV is under the spectral resolution of the setup, while based on the XX fitting FSS $= 39.2 \pm 3.4 \mu$eV. d) Time-resolved photoluminescence, decay times from the fit: $\tau_1 = 1.33 \pm 0.06$ ns and $\tau_2 = 5.8 \pm 1.4$ ns.

[3] H. Y. Kim, M.-Y. Ryu, J. S. Kim, *Journal of Luminescence* **2012**, *132*, 7 1759.

[4] T. Hakkarainen, J. Hilska, A. Hietalahti, S. Ranta, M. Peil, E. Kantola, A. Chellu, E. Sen, J.-P. Penttinen, M. Guina, Telecom wavelength single-photon source based on ingasb/algasb quantum dot technology, **2024**.

[5] J. Skiba-Szymanska, R. M. Stevenson, C. Varnava, M. Felle, J. Huwer, T. Müller, A. J. Bennett, J. P. Lee, I. Farrer, A. B. Krysa, P. Spencer, L. E. Goff, D. A. Ritchie, J. Heffernan, A. J. Shields, *Phys. Rev. Appl.* **2017**, *8* 014013.

[6] D. Gammon, E. S. Snow, B. V. Shanabrook, D. S. Katzer, D. Park, *Science* **1996**, *273*, 5271 87.

[7] G. Moody, M. E. Siemens, A. D. Bristow, X. Dai1, A. S. Bracker, D. Gammon, S. T. Cundiff, *Phys. Rev. B* **2011**, *3* 245316.

[8] D. G. Nahri, C. H. R. Ooi, *J. Opt. Soc. Am. B* **2014**, *31*, 5 1182.
